# Cartilage Tissue Engineering Approaches Need to Assess Fibrocartilage When Hydrogel Constructs Are Mechanically Loaded

**DOI:** 10.3389/fbioe.2021.787538

**Published:** 2022-01-12

**Authors:** Hamed Alizadeh Sardroud, Tasker Wanlin, Xiongbiao Chen, B. Frank Eames

**Affiliations:** ^1^ Division of Biomedical Engineering, College of Engineering, University of Saskatchewan, Saskatoon, SK, Canada; ^2^ Department of Anatomy, Physiology, and Pharmacology, University of Saskatchewan, Saskatoon, SK, Canada; ^3^ Department of Mechanical Engineering, College of Engineering, University of Saskatchewan, Saskatoon, SK, Canada

**Keywords:** cartilage tissue engineering, mechanical compression, *in vitro*, *in vivo*, Col1, Col2, hyaline, fibrocartilage

## Abstract

Chondrocytes that are impregnated within hydrogel constructs sense applied mechanical force and can respond by expressing collagens, which are deposited into the extracellular matrix (ECM). The intention of most cartilage tissue engineering is to form hyaline cartilage, but if mechanical stimulation pushes the ratio of collagen type I (Col1) to collagen type II (Col2) in the ECM too high, then fibrocartilage can form instead. With a focus on Col1 and Col2 expression, the first part of this article reviews the latest studies on hyaline cartilage regeneration within hydrogel constructs that are subjected to compression forces (one of the major types of the forces within joints) *in vitro*. Since the mechanical loading conditions involving compression and other forces in joints are difficult to reproduce *in vitro*, implantation of hydrogel constructs *in vivo* is also reviewed, again with a focus on Col1 and Col2 production within the newly formed cartilage. Furthermore, mechanotransduction pathways that may be related to the expression of Col1 and Col2 within chondrocytes are reviewed and examined. Also, two recently-emerged, novel approaches of load-shielding and synchrotron radiation (SR)–based imaging techniques are discussed and highlighted for future applications to the regeneration of hyaline cartilage. Going forward, all cartilage tissue engineering experiments should assess thoroughly whether fibrocartilage or hyaline cartilage is formed.

## Introduction

In osteoarthritis (OA), articular cartilage at the end of bones, such as in the knee or elbow joint, degrades by various injuries or normal wear and tear caused by aging. In addition, cartilage damage can affect the mobility of injured young patients (Buckwalter and Mankin, 1998). Articular cartilage is hyaline cartilage, an avascular tissue that contains chondrocytes within a dense extracellular matrix (ECM) mainly composed of proteoglycans and collagen type II (Col2) (Landinez et al., 2009; Becerra et al., 2010). Since hyaline cartilage does not have self-healing potential, various clinical strategies, such as partial or total joint replacement, microfracture, and chondrocyte implantation, have been developed and employed to treat damaged articular cartilage. However, these treatments do not result in ever-lasting outcomes, and patients typically need secondary surgeries. Relevant to this review article, another challenge of these treatments is that they can lead to formation of fibrocartilage, which has inferior mechanical properties to hyaline cartilage in carrying out the function of articular cartilage (Li K et al., 2017).

To provide a more permanent solution to articular cartilage damage, cartilage tissue engineering (CTE) is an interdisciplinary approach that combines knowledge of engineering and cell biology. In this regard, various synthetic and natural biomaterials with proper biocompatibilities have been used to fabricate hydrogel, polyester-based solid, and/or hybrid constructs (Little et al., 2011; Olubamiji et al., 2016a; Olubamiji et al., 2016b; You et al., 2016; You et al., 2017; Armiento et al., 2018; You et al., 2018; Sadeghianmaryan et al., 2020). Hydrogels have received much interest as scaffolds for CTE studies because similar to native cartilage, cells can be impregnated within hydrated polymer networks to maintain chondrocyte morphology and phenotype (Vega et al., 2017).

A major limitation, however, is that hydrogels do not have adequate mechanical strength to withstand applied forces after *in vivo* implantation, and this force application can change the type of cartilage formed. Applied forces are transmitted to the impregnated cells, which respond by expressing such proteins as Col1 and Col2 (Bian et al., 2010; Nebelung et al., 2012). The presence of Col1 is a big problem for articular cartilage regeneration because hyaline cartilage has little to no Col1, whereas Col1 is a marker of fibrocartilage (Ham, 1965). Bioreactors have been utilized *in vitro* to simulate the joint’s compressive forces *in vivo*. To various levels of success, mechanical stimulation of hydrogels promoted some biological activities of chondrocytes, including non-specific measures of collagen secretion (Schulz and Bader, 2007; Natenstedt et al., 2015). However, a critical aspect of such studies is to see what type of collagens was produced in response to compression forces *in vitro*. If the ratio of Col1:Col2 is relatively high, then the experiment produced a more fibrocartilage-like tissue, which has inferior biological and mechanical properties to hyaline cartilage when seeking to regenerate articular cartilage.

Whether implanted hydrogels work for hyaline cartilage regeneration *in vivo*, where various physiological and mechanical factors are involved, needs to be investigated before going to clinical applications (Chu et al., 2010). To be fair, the *in vivo* mechanical environment of joints is extremely complex, making it extremely difficult to simulate using a bioreactor. The implantation of hydrogel constructs within the joints of various animal models needs to be investigated to see whether the resulting tissue formed is the desired hyaline cartilage or fibrocartilage. Again, the ratio of Col1:Col2 produced in these experiments is critical, so we begin by reviewing how current *in vitro* and *in vivo* studies have analyzed specific collagen expression in determining whether hydrogel-loaded constructs produce hyaline cartilage or fibrocartilage.

In almost every experimental study to date, the implanted constructs need to be extracted by invasive and destructive techniques after euthanizing the animals in order to evaluate the type of cartilage formed. Different visualizing tools can also be utilized to assess tissue regeneration and construct degradation without the need to sacrifice the animals (Izadifar et al., 2016b; Ning et al., 2021). By using the novel imaging techniques covered in this review, the number of required animals for preclinical experiments would be reduced, and these methods can be adapted for future clinical applications.

Cells respond to applied mechanical forces by varying biochemical signals that can affect gene expression within the cells (Knobloch et al., 2008). Some CTE studies illustrated that compressive force applied to mono-layer chondrocytes or 3D chondrocyte-impregnated hydrogels activate cellular signaling pathways (Allen et al., 2012; Bougault et al., 2012; Sanz-Ramos et al., 2012). Several signaling mechanisms are involved in mechanical stimulation of chondrocytes, and their influence on gene expression will be also reviewed in this article.

As an overview for this article, mechanical compressive loading can play a crucial role in the regeneration of new hyaline cartilage within hydrogel constructs by stimulating cells to express collagen genes and depositing these collagens to the ECM. The cartilage in joints is abundant in Col2, but deposition of Col1 from the impregnated cells can lead to a fibrocartilage-like tissue with inferior mechanical and biological properties compared to native articular cartilage. Thus, the generation of Col1 and Col2 in mechanically loaded, chondrocyte-impregnated hydrogels is the focus of this article.

### Hyaline vs. Fibrocartilage: Cellular, Molecular, and Mechanical Differences

Hyaline cartilage is a shiny, white, translucent, and flexible cartilage and can be found in the articular surfaces of movable joints such as knee and elbow. Other than joints, hyaline cartilage is also present in the rib tips, nose, larynx, and the rings of the trachea (Parsons, 1998). Cell types do differ between hyaline cartilage and fibrocartilage, but they are two types of chondrocytes: one is called a hyaline matrix–rich chondrocyte, and the other is a cell-rich fibrous chondrocyte (Benjamin and Ralphs, 1991). Articular cartilage has a very low cell density (5% of cartilage mass), and hyaline matrix–rich chondrocytes are surrounded by the cartilage ECM either as single cells or as clusters of cells (Anderson et al., 1964). In total, 30% of the hyaline cartilage ECM by weight is a solid component that is rich in ground substance including mostly glycosaminoglycans (GAGs) and Col2 fibers. Sulfation of GAGs in the ECM attracts water, which makes up 70% of the ECM of hyaline cartilage by weight and gives articular cartilage its tremendous compressive resistant strength (Brown and Eames, 2016). Articular cartilage has four different zones, including a superficial zone, a middle or transitional zone, a deep zone, and a calcified zone. Contents of the GAGs and collagens vary from the superficial zone to the calcified zone. The collagen content decreases from the top to the bottom of the articular cartilage, whereas the GAGs increase in this direction. Apart from the very thin superficial zone, the other zones are types of hyaline cartilage. The mechanical properties of articular cartilage also vary according to the zonal organization of the ECM molecules, and the compressive modulus increases from the superficial zone to the calcified zone of the articular cartilage (Schinagl et al., 1997).

Fibrocartilage is a white, dense, opaque, and flexible cartilage located in the intervertebral discs of the spine, tendons, ligaments, and jaw (Benjamin and Ralphs, 2004). Cell density within fibrocartilage is higher than that in hyaline cartilage, and the two major types of cells in fibrocartilage are chondrocytes and fibroblasts. Single or very small groups of fibroblasts and cell-rich fibrous chondrocytes are in lacunae, and their shape can be round, but most fibrocartilage lacunae are axially aligned with the collagen fibers (Benjamin and Evans, 1990). In contrast to hyaline cartilage, fibrocartilage has abundant Col1 in addition to Col2. Fibrocartilage contains denser and spatially organized collagen fibers than hyaline cartilage, so fibrocartilage is the strongest type of cartilage in the body (Poole et al., 1982; Kheir and Shaw, 2009). Critically for current clinical problems in articular cartilage regeneration, fibrocartilage also has very low levels of GAGs, which means that it lacks the compressive resistant force needed at surfaces of articulating joints (Armiento et al., 2019).

Table 1 shows the differences between hyaline cartilage and fibrocartilage. The main difference between them is the high level of Col1 in fibrocartilage, whereas Col1 is very low or absent in hyaline cartilage. Also, the different ECM features of hyaline and fibrocartilage give them different mechanical features from each other. Hyaline cartilage has high compressive and low tensile properties, whereas fibrocartilage has low compressive and high tensile properties.

**TABLE 1:** Summary of characteristics of hyaline and fibrocartilage.

	Hyaline	Fibrocartilage
Location	Joints, rib tips, nose, larynx, and the rings of the trachea	Intervertebral discs of the spine, tendons, and ligaments and jaw
Appearance	Shiny, white, and translucent	White, dense, and opaque
Cell type	Hyaline matrix–rich chondrocytes	Cell-rich fibrous chondrocytes and fibroblasts
Cell organization	Round single or cluster of cells in lacunae	Single and small groups of cells in lacunae, round or aligned in rows
ECM	GAGs and Col2	GAGs, Col1, and Col2

### *In Vitro* Bioreactors Can Mimic *In Vivo* Compression Forces

Mechanical forces at an appropriate magnitude in a physiological range are essential for the maintenance of hyaline cartilage to prevent its degeneration. These forces stimulate chondrocytes for the biosynthesis of cartilaginous molecules needed for the integrity and maintenance of cartilage. However, over-loading can result in cartilage damage and degenerative joint diseases (Musumeci, 2016). Native cartilage in joints endures different mechanical forces without getting damaged, and hence fabricated cartilage constructs must withstand similar mechanical forces meanwhile generating and maintaining hyaline cartilage.

In this regard, biodynamic machines have been utilized as *in vitro* bioreactors to culture cell-impregnated hydrogels in order to understand how cells might respond to the physiological or superphysiological loadings that they might encounter if they were implanted *in vivo*. However, many types of force exist in joints, and simulating all those forces in a single bioreactor is not feasible (Wimmer et al., 2004; Grad et al., 2011). Hence, most of the custom-made or commercial bioreactors have been developed to apply a single type of force, such as compression (Kisiday et al., 2004; El-Ayoubi et al., 2011), tension (Lee J. K et al., 2017; Wu S et al., 2017), or shear (Marlovits et al., 2003; Gemmiti and Guldberg 2009). Multi-force bioreactors also have been developed to include two types of force, such as compression and linear shear stress, in a bioreactor chamber (Marlovits et al., 2003; Waldman et al., 2007). Compressive loading is one of the major mechanical forces applied on articular cartilage (Martel-Pelletier et al., 2008), and accordingly, most *in vitro* studies investigated the responses of cells to compressive forces (Hunter et al., 2002; Bryant et al., 2008; Pelaez et al., 2009; Wang et al., 2009).

Hydrogels are a popular type of constructs for CTE applications and have been used extensively for *in vitro* mechanical compressions and *in vivo* implantations (Bryant et al., 2008). These constructs are soft polymeric networks with low mechanical properties. Hydrogels will never mimic cartilage ECM because at most a hydrogel has a few 100 kPa compression modulus that is 0.03–1% of native cartilage having a compression modulus of 0.08–320 MPa increasing from the superficial to the calcified zone (Schinagl et al., 1997; Yang and Temenoff, 2009; Karimi et al., 2015). The effects of mechanical compression on chondrocytes impregnated within hydrogels both *in vitro* and *in vivo* will be discussed in the following sections.

### Static Compression Suppressed Cartilage Regeneration

Application of a compression force can be in a static force regime by which a constant compressive strain is subjected to the engineered constructs for a continuous and limited time. Compressive strain is the deformation of the constructs in one spatial dimension due to the applied compressive force. Various studies applied static compression loadings on cartilage explants and engineered cartilage constructs. However, this type of force regime did not show satisfactory results of cartilage growth and even inhibited the secretion of cartilage ECM (Jones et al., 1982; Gray et al., 1988; Mouw et al., 2007). For example, bovine cartilage explants were compressed for 24 h, and as a result, transcription levels for *Col2a1* and *aggrecan* were down-regulated compared to those of unloaded samples (Fitzgerald et al., 2004). Chondrocytes seeded on non-woven polyglycolic acid (PGA) composites and subjected to 50% static compression for 24 h resulted in 35 and 57% reduction in total protein and sulfated GAG secretion, respectively (Davisson et al., 2002). Static compression of agarose gels did not change the biosynthesis of cartilage over short periods of loading but did reduce cartilage production after longer durations of compression (Buschmann et al., 1995). 25 and 50% static compression of chondrocytes cultured in Col1 hydrogels inhibited both *Col1a2* and *Col2a1* expressions after 24 h (Hunter et al., 2002). ECM pore sizes of cells might be reduced due to static compression when they are impregnated in hydrogels. Consequently, nutrient transportation into cells is reduced, suppressing the expression of cartilage ECM (Freeman et al., 1994; Guilak et al., 1995). Such widely reported inhibitory effects of static compression on chondrocytes led most researchers to switch to dynamic compression force applications.

### Dynamic Compression Showed Limited Positive Effects on Cartilage Regeneration

In the natural environment of a joint, articular cartilage is subjected to dynamic forces during walking, running, and jumping motions (Chen C et al., 2013). Therefore, recent studies subjected hydrogel constructs to dynamic forces in order to observe the response of chondrocytes (Sauerland et al., 2003; Martel-Pelletier et al., 2008; Ryan et al., 2009).

Two major parameters within dynamic compression systems are the magnitude of compressive strain and the duration of loading. Human joints undergo cartilage deformations during physiological movements. Various techniques have estimated the applied physiological strains, when human joints move, to be in a range of 3–20% cartilage thickness deformation (Armstrong et al., 1979; Macirowski et al., 1994; Eckstein et al., 1999; Eckstein et al., 2000). Consequently, CTE *in vitro* compression studies worked in this strain range, mostly using 10% strain as a simulated physiological strain (Mauck et al., 2000; Mauck et al., 2003; Lima et al., 2007).

The GAG content was commonly analyzed in samples subjected to physiological compression strains, but unfortunately, specifying the levels of Col1 and Col2 production, which would be required to analyze fibrocartilage formation, were neglected in most studies (Mauck et al., 2000; Shelton et al., 2003; Kisiday et al., 2004). For example, chondrocyte-impregnated agarose hydrogels were subjected to 3% dynamic strain with different frequencies ranging from 0.001 to 1.0 Hz for different loading durations. Higher DNA and GAG accumulation were reported at 23 days (a relatively late timepoint) in the dynamically loaded samples (Buschmann et al., 1995). Assessment of hydroxyproline to indicate total collagen content was performed on chondrocytes seeded in agarose, fibrin, or peptide hydrogels that were loaded with different strains (2.5–14%) (Mauck et al., 2000; Mauck et al., 2003; Hunter et al., 2004; Kisiday et al., 2004). For example, chondrocyte-impregnated agarose gel discs were dynamically loaded using a custom-designed bioreactor at a frequency of 1 Hz and 10% strain for 4 weeks. Both GAG and hydroxyproline contents were greater for the loaded samples than unloaded controls at day 21 (Mauck et al., 2000). Initial cell density in these experiments also positively influenced both mechanical properties and cartilage tissue growth within the 10% strain-loaded hydrogels. The dynamic force regime had 1 Hz frequency and was applied 2 h per day and 5 days per week. Enhancement in GAG and collagen content (∼150%) and mechanical properties (∼2 fold) were observed with 10 × 10^6^ cells/ml. Mechanical properties were improved compared to the unloaded group, especially when using a higher cell density, but GAG and collagen contents were the same for loaded and unloaded samples (Mauck et al., 2003). The total collagen content could be indirectly quantified through hydroxyproline content; however, this assessment does not reveal how much Col1 vs. Col2 was produced in the loaded constructs.

In contrast, a few studies assayed Col1 and Col2 specifically within loaded constructs in the range of physiological strain (∼10%). *Col2a1* promoter activity was decreased when chondrocyte-impregnated agarose hydrogels were subjected to 10% strain dynamic loading at 1 Hz frequency for a relatively short experimental time of 3 h (Mauck et al., 2007). Chondrocytes impregnated in photopolymerized methacrylated hyaluronic acid (HA) constructs were subjected to 10% strain dynamic loading with a frequency of 1 Hz for 1 and 5 days (Chung et al., 2008). Compared to the unloaded group, loaded samples upregulated both *Col1a1* and *Col2a1* gene expression and had an increased *Col2a1/Col1a1* ratio. However, these samples were loaded for relatively short time periods compared to the *in vivo* situation, which subjects constructs to loading for many years, if not decades.

Long-term loading better reflects *in vivo* conditions, but most studies on chondrocyte-impregnated hydrogels loaded for a relatively long time only reported Col2 production, disregarding assessments for Col1. This is a huge gap in current CTE compression studies. However, data are still useful on how cells in a hydrogel environment respond to loading by producing Col2, but whether these data reflect hyaline cartilage or fibrocartilage formation is an unanswered question. For example, stronger Col2 staining was seen in agarose constructs that underwent long-term loading (Kelly et al., 2008; Ng et al., 2009). Brighter staining of GAGs, Col2, and aggrecan was observed in alginate hydrogels impregnated with osteoarthritic chondrocytes that were dynamically compressed with 15% strain at 1 Hz over 2 weeks, than that in unloaded samples (Jeon et al., 2012). However, other studies showed decreased Col2 production. For example, Col2 deposition was reduced in both juvenile and adult chondrocyte-impregnated poly (ethylene glycol) (PEG) hydrogels that were subjected to long-term (14 days) dynamic compressive loading at 1 Hz and 10% strain (Farnsworth et al., 2013). Unfortunately, there was no assessment for Col1 deposition in this study.

In a bad omen for hydrogel-only CTE approaches, several studies showed that long-term dynamic loading of hydrogel constructs stimulated chondrocytes to produce fibrocartilage-like tissue, such as a reduction in Col2 or increase in Col1 production. Long-term dynamic loading of PEG hydrogels with 15% strain compression at 1 Hz frequency upregulated *Col1a2* gene expression, relative to the unloaded group, but these expression levels returned to that seen in the unloaded group at a later timepoint. On the other hand, *Col2a1* expression did not change in the loaded group in this study over 7 days of compression (Bryant et al., 2008). Higher accumulation of Col2 was observed in Col1 hydrogels loaded with 10% strain at 0.3 Hz than that in the unloaded samples after 28 days of loading (Nebelung et al., 2012). However, no significant difference in the *Col2a1*/*Col1a1* ratio was found between loaded and unloaded samples, and this ratio was less than one for the loaded constructs. A Col2/Col1 ratio less than one indicated the formation of a fibrocartilage-like tissue under this applied physiological strain.

For *in vitro* studies that loaded hydrogel constructs, the lack of assessing both Col1 and Col2 is very problematic. However, the general outcome could be that short-term loading is useful for upregulation of Col2, although these experiments do not simulate long-term force application experienced in articular cartilage *in vivo*. When both Col1 and Col2 were analyzed, current long-term *in vitro* loading studies of chondrocyte-impregnated hydrogel constructs suggested that fibrocartilage was forming instead of hyaline cartilage. Specifically, long-term loading of hydrogels did not result in higher Col2 and/or lower level of Col1 production. Table 2 summarizes key findings and characteristics of different *in vitro* compression experiments on chondrocyte-impregnated hydrogel constructs.

**TABLE 2:** Summary of information for various *in vitro* compression strategies.

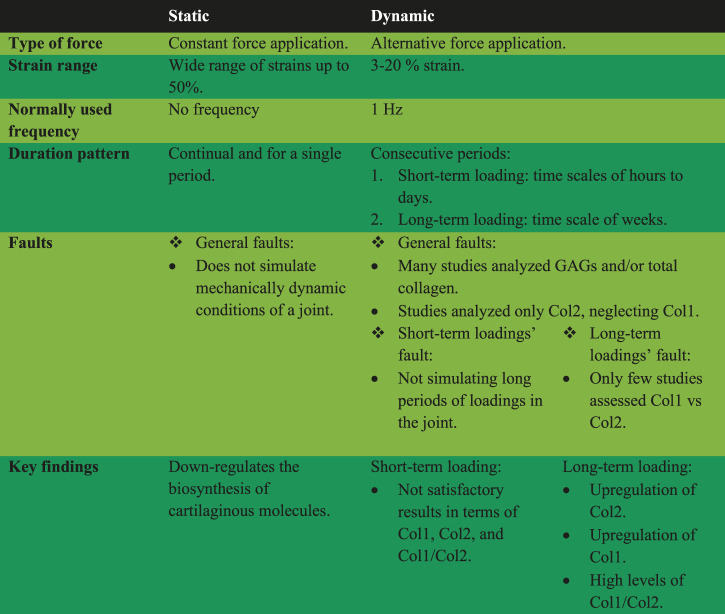

### Implantation Studies Can Elucidate Function of Hydrogels in *In Vivo*

#### Animal Models for Articular Cartilage Regeneration

Examining the *in vivo* function of fabricated hydrogels within animal models is essential before moving forward to clinical applications because these experiments test the performance of CTE constructs in a preclinical setting. Implantation of constructs into joints is much better than other sites, such as subcutaneous implantations to mimic the force environment of future clinical applications; however, studies investigating the formation of cartilage in subcutaneously implanted hydrogels are a useful first step for observing the function of chondrocyte-impregnated constructs *in vivo*. Performing an *in vivo* CTE study requires consideration of several different parameters, such as the size and weight of the animal, joint size, cartilage thickness, load distribution within the joint, costs, convenience of the operation, and animal handling (Malda et al., 2013; Moran et al., 2016). Animals used for implantation of hydrogel CTE constructs generally fall into two categories, small and large models, and multiple types of animals from each group will be discussed below.

Rodents such as mice and rats are cost-effective small animal models that are also easy to breed and house. However, cartilage implantation in joints of these animals has been rarely carried out because the very small joint size makes it difficult to perform an operation (Gelse et al., 2003; Kuroda et al., 2006). Mice and rats also have very thin cartilage with thin layers of chondrocytes, so the outcomes of implantation might not predict human applications. Nevertheless, mouse models have been used for subcutaneous and intramuscular implantation of hydrogel constructs for 6–8 weeks. These implantation sites might be useful for testing the biodegradation of the hydrogels as well as cartilage matrix formations *in vivo*, although they do not provide mechanical conditions existing in joints that might influence these parameters (Haisch et al., 2000a; AMIEL et al., 2001).

Rabbits have been used extensively for research in the CTE field and implantation of tissue-engineered constructs (Chu et al., 2010). Similar to mice and rats, rabbits are also affordable and easy to breed and house. The joint size of rabbits is larger than that of other small animals, which makes the surgery procedures easier (International, 2005). Rabbits have relatively thicker cartilage (0.3–0.44 mm) than mice and rats (∼0.3 mm), although it is still much thinner than human cartilage (∼2.35 mm) (Räsänen and Messner, 1996; Frisbie et al., 2006; Vos et al., 2012). Rabbit cartilage showed great endogenous repair in articular defects compared to that of other animals and humans (Hunziker, 1999). The very low self-healing of human cartilage is related to low cell densities (1,800 cells/mm^3^), whereas higher cellularity of the rabbits (7,500 cells/mm^3^) contributes to more endogenous cartilage repair (Moran et al., 2016).

The sheep is a commonly used large animal model that has similar joint anatomy to the human joint. These animals are easily accessible, cost-effective, and also easy to handle and house. However, they have thinner cartilage (0.4–0.5 mm) than the human cartilage, thus created defects reaching to the subchondral bone region in most studies (Lu et al., 2000; Frisbie et al., 2006). Despite the mentioned disadvantages, sheep can be an appropriate large animal model for assessment of hydrogels in mechanically loaded conditions.

Goats are popular large animal models for cartilage implantation (Brehm et al., 2006; Lind et al., 2008; Marmotti et al., 2013). Their large joint size allows creating large defects in articular cartilage that are unable of spontaneous healing (Jackson et al., 2001; Ahern et al., 2009). The cartilage thickness is around 1.1 mm in goats, greater than that in sheep, but it is still lower than human cartilage thickness (Jackson et al., 2001). Although defects of 12 mm diameter can be created in goats, commonly created defects are smaller than the natural defects in humans. Generally, goats are proper large animal models for the assessment of implants in small cartilage defects (Ahern et al., 2009).

A good example of a large and robust animal model is the horse, and this animal is similar to the human in terms of different aspects of the joint characteristics. Their joint is very big with similar anatomy to the human joint, and they have a thick cartilage region (1.75 mm) (Frisbie et al., 2006; Moran et al., 2016). Cartilage and osteochondral defects with various thickness and diameter can be created in horses because of their great cartilage size. However, their large weight of 400–600 kg causes rigorous loading forces on cartilage (Ahern et al., 2009; Chu et al., 2010). High mechanical forces on the joint, large and expensive facilities for housing and breeding, and specialized personnel and equipment are important factors to be considered for carrying out cartilage surgeries on horses (Ribitsch et al., 2020).

Pig models offer several advantages for cartilage surgeries. Mature pigs have a wide range of weight up to 250 kg, and they mimic human joints in terms of size, mechanical loading, and cartilage thickness (1.5–2 mm), which allows the creation of chondral and osteochondral defects with various sizes. However, their housing and performing operations on them are very costly. Besides, specialized facilities and surgeons are required to perform the surgeries and afterward housing. Nevertheless, if a group of experts could overcome the costs and needs for pig surgery, this animal is an appropriate model for cartilage repair studies (Frisbie et al., 2006; Chu et al., 2010; Meng et al., 2020). On the other hand, many studies have performed surgeries on mini-pigs weighing 40–70 kg to overcome issues with larger pigs, although lower loading forces are on the joints of mini-pigs due to their lighter weight (Christensen et al., 2015).

**FIGURE 1:**
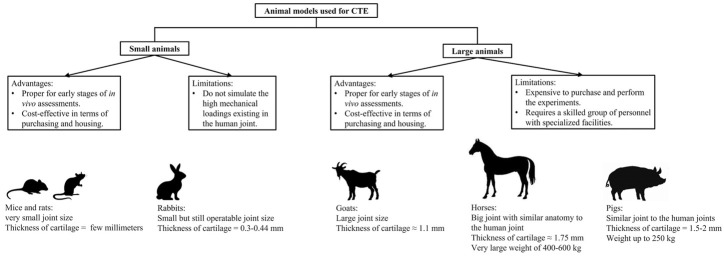
Summary of information for advantages and limitations of small and large animal models used in CTE. Specific characteristics for several animal models are also presented in the figure (images for this part were generated from https://www.istockphoto.com).

Selection of an appropriate animal model is crucial to relate the results of an implantation study to clinical settings. In this regard, small animal models, such as rats and rabbits, seem to be good candidates because they are cost-effective in purchasing and housing. The results cannot be correlated to clinical applications because of the various disadvantages mentioned previously. However, they can be used for early stages of assessments such as biodegradation and biocompatibility of the tissue-engineered hydrogels as well as the formation of cartilage ECM *in vivo*. On the other hand, large animals, such as pigs and horses, are more appropriate models for pretesting implants before clinical trials because of the high similarity of their joints to human joints in size, cartilage thickness, joint mechanics, and magnitude of applied forces on cartilage. Summary of this section is illustrated in Figure 1 with the advantages and limitations related to small and large animal models.

### Small Animal Models Are Useful for Initial *In Vivo* Experiments

Different types of hydrogels such as agarose (Armiento et al., 2018), fibrin (Westreich et al., 2004), alginate (Paige et al., 1996; Eslaminejad et al., 2007; Liao et al., 2017), HA (Park et al., 2019), composite hydrogels (Li T et al., 2019) such as chitosan and chondroitin sulfate (CS) (Li C et al., 2019) and also novel biomaterials such as sericin methacryloyl (Qi et al., 2018) have been implanted subcutaneously in mouse and rabbit models. Despite the absence of joint mechanical conditions, subcutaneous investigations are important and appreciated in terms of evaluating hydrogel constructs under a physiological *in vivo* condition.

Most of the subcutaneous implantation studies only assessed cartilage formation by histology and gross observations (Chen et al., 2017). For example, polymerized alginate was used as an injectable gel for cartilage formation, and chondrocyte-impregnated alginate hydrogels were injected subcutaneously into a nude mouse (Paige et al., 1996). Gross and histology observations showed the formation of cartilage-like tissues at 8 and 12 weeks post implantation, but neither the deposition of aggrecan nor collagen molecules were analyzed. Formation of cartilage ECM histology was also reported by implantation of a fibrin hydrogel in rabbit and mouse models (Sims et al., 1998; Westreich et al., 2004). Cartilage formation was reported in 85% of fibrin samples injected into subcutaneous sites of rabbits, and the newly formed tissue appeared like cartilage, from the gross and histological aspects (Westreich et al., 2004). Although these studies reported the formation of cartilage ECM in implanted hydrogels, the assessments were not sufficient, and further analyses must be carried out to determine the type of cartilage formed.

Some studies assessed Col2 deposition within implanted hydrogels (Choi et al., 2007; Yun and Moon, 2008; Öztürk et al., 2020), although few of them performed assessments for production of both Col1 and Col2. For instance, chondrocyte-impregnated recombinant collagen hydrogels supported neocartilage formation by deposition of Col2, although Col1 was also present in the implanted constructs (Pulkkinen et al., 2010). Gelatin methacrylate (GelMA) and glycidyl methacrylate-modified GelMA (GelMAGMA) hydrogels showed the presence of Col2 and slight deposition of Col1 in the dorsa of mice 6 weeks after implantation (Li X et al., 2017).

Subcutaneous implantation is an easier surgery than joint surgeries, but the mechanical environment in the joint is worth performing surgeries and observing the functions of cells in response to forces. After completion of any *in vivo* joint experiment, animals are euthanized, joint samples are extracted, and then sections are generated from OCT or paraffin-embedded explants for further histological and immunohistochemical assessments. Many joint implantation studies focused on evaluating the formation of glycosaminoglycans and collagen fibers by histology assessments (Fragonas et al., 2000; Holland et al., 2005; Kim et al., 2013). Alginate is a popular biomaterial that has been extensively used for cartilage regeneration (Wong et al., 2001; Balakrishnan and Banerjee, 2011). As an example, autologous nasal chondrocytes were impregnated in alginate hydrogels and implanted into rabbit joints. Repaired hyaline cartilage–like tissue was reported in osteochondral defects of the trochlear groove based on histological staining (Chen et al., 2018). Self-settling cellulose-based hydrogels that were impregnated with nasal chondrocytes showed no signs of inflammation 6 weeks after implantation into rabbit knees, and cartilage matrix formed, based on histology and Col2 immunohistochemistry (Vinatier et al., 2009). In a recent study, a novel injectable alginate hydrogel containing porous polymeric microspheres with calcium gluconate as a cross-linker showed evidence of cartilage repair after implantation into the patellar groove of rabbit knees (Liao et al., 2017). Specifically, GAGs and Col2 were highly expressed, and the repaired cartilage appeared to integrate with host cartilage. However, Col1 production was not assessed to determine whether the new cartilage is hyaline cartilage or fibrocartilage.

Necessary assessments of Col1 production must be performed by some sort of immunological analysis in order to assess hyaline cartilage vs. fibrocartilage (Zhao et al., 2015), and only a few studies so far have done so. Chondrocyte-impregnated recombinant collagen hydrogels were implanted into osteochondral defects in the femoral trochlea of rabbits (Pulkkinen et al., 2013). The deeper parts of the defects contained Col2 after 6 months, while Col1 was mostly present in the superficial regions. In a recent study, two types of fibrin gels were implanted into rabbit joints with either redifferentiated or dedifferentiated chondrocytes, and both Col1 and Col2 depositions were tested in the implants. After 6 weeks of implantation, the outcome was more of a fibrocartilage-like tissue for both types of constructs, although deposition of Col2 was still higher than that of Col1. The ratio of Col2/Col1 was reported as 2.8 and 2.1 for dedifferentiated and redifferentiated constructs, respectively (Bianchi et al., 2019). According to these studies, Col2 formation has been generally reported to be high in the implanted defects, but the formation of Col1 was still observed within the defects. Despite this general outcome, a couple of studies reported low Col1 production. For example, Col1 synthesis was not seen in chondrocyte-impregnated collagen type II hydrogels 24 weeks after implantation in rabbit joints (Funayama et al., 2008).

### Large Animal Studies Lack Col1 vs. Col2 Assessment for Implanted Hydrogels

Implantation in large animal models can provide a better sight of how the fabricated hydrogels regenerate cartilage in a mechanically analogous environment to human joints. Joints of large animals exhibit multiple mechanical forces, such as compression, sheer, and tension, that are similar to human joints in terms of magnitude and type. Simulating all mechanical forces existing in the joint *in vitro* is not feasible; hence, it is essential to perform hydrogel implantation in the joints of large animals. However, not many published studies performed hydrogel implantations in large animals, which could be due to technical and economic difficulties related to the surgeries. Studies that implanted the hydrogel in large animals allowed them to walk freely with force loading on all joints. Various assessments have been used to analyze the extracted joint samples, and some have only performed histology analyses. Also, various biomaterials have been used for implantation in different animal models. Therefore, this section will review different hydrogels based on the types of biomaterial used (Liu et al., 2002; Sanz-Ramos et al., 2014).

Fibrin has been a commonly used biomaterial for impregnating chondrocytes and cartilage regeneration within large animal defects, including horses, goats, and pigs (Hendrickson et al., 1994; Van Susante et al., 1999; Peretti et al., 2006). For example, xenografted rabbit chondrocytes were impregnated in fibrin glue and implanted in adult goats. Xenografted defects showed initially better cartilage regeneration, but after 1 year, both grafted and control defects were similar and filled with fibrocartilaginous tissue. After 3 weeks of implantation, 17% of total collagen in the grafted constructs was Col2, and while this number was enhanced to 75% after 1 year, Col1 was not analyzed (Van Susante et al., 1999). In another study, an osteochondral defect in a goat model was treated with a double-phase construct comprised chondrocyte-impregnated fibrin glue upon a hydroxyapatite cylinder (van Susante et al., 1998). Repaired tissue was reported as fibrocartilage based on histology. Nevertheless, several animal studies have reported immunological responses from the host animals to exogenous fibrin implants (Kawabe and Yoshinao, 1991; Haisch et al., 2000b).

Alginate has been also used in several *in vivo* implantation studies for CTE applications (Wong et al., 2001; Balakrishnan and Banerjee, 2011; Farokhi et al., 2020). Chondrocytes and stem cells from different sources that have been cultured within the 3D matrix of alginate hydrogels have synthesized components of cartilage matrix. Despite these findings, few studies implanted alginate hydrogel constructs into the joints of large animal models (Hauselmann et al., 1996; Diduch et al., 2000; Mierisch et al., 2003; Almqvist et al., 2009). Osteochondral defects of sheep joints treated with chondrocyte-impregnated alginate spheres showed no histological sign of cartilage formation 21 days after implantation, containing fibrous tissue with fibroblast-like spindle cells and no safranin O staining (Heiligenstein et al., 2011). Alginate–gelatin composite hydrogels containing periosteal cells and chondrocytes implanted in sheep joints showed higher levels of Col2 and perhaps lower levels of Col1, than the untreated defects (Schagemann et al., 2009). However, Col1 intensity was very close to Col2 in the implanted groups, much higher than that in normal hyaline cartilage, indicating that the newly formed cartilage was likely fibrocartilage.

Collagen has been also used for different tissue engineering applications, including CTE, despite being expensive and having low availability and mechanical properties (Galois et al., 2006). Chondrocyte-impregnated collagen hydrogels were implanted in trochlear defects of a canine model, and fibrous and fibrocartilage tissues were formed within the defects (Nehrer et al., 1998). Regeneration of cartilage was improved by cell-impregnated collagen hydrogels in sheep knees, but the conclusions were only based on histology and quantifications with the Mankin score without any assessments for Col1 and Col2 (Sanz-Ramos et al., 2014). Chondrocytes and bone marrow stromal cells (BMSCs) seeded onto bi-layered collagen matrices formed a lower layer of Col1 and Col3 as a somewhat mechanically stable base, and the upper layer consisted of Col2 (Dorotka et al., 2005). The outcome was greater repair and hyaline-like cartilage regeneration for the seeded implants with a microfracture treatment, based on histology, and Col1 and Col2 staining. Chronologically predifferentiated mesenchymal stem cells (MSCs) impregnated within Col1 hydrogels and implanted in sheep showed better cartilage repair for the predifferentiated MSCs than for chondrocytes in terms of histology and Col2 staining (Marquass et al., 2011). Col1 staining was pretty high in all experimental hydrogel groups, although the article related that to immunoreaction with the Col1 hydrogel itself.

CS, a polysaccharide biomaterial that is a major component of cartilage ECM, has shown beneficial effects for cartilage formation both *in vitro* and *in vivo* (Ronca et al., 1998; Li et al., 2004; Chen W. C et al., 2013). However, similar to all hydrogels, pure CS exhibits low strength as a scaffold and degrades very fast, making *in vivo* application very challenging (Chang et al., 2010). Using CS as a bioadhesive for defects created in goat femoral condyles, cell-free poly (ethylene glycol) diacrylate (PEGDA) hydrogels were injected into the defects with marrow stimulation (Wang et al., 2007). The results showed that safranin O staining was quantitively higher for the hydrogel-treated defects than that for the untreated defects.

Chitosan is a natural polysaccharide and partially de-acetylated derivative of chitin that has structural similarity to the native GAGs present in the cartilage (Suh and Matthew, 2000). Chondrocyte-impregnated chitosan hydrogels implanted in sheep knees showed formation of hyaline-like cartilage after 24 weeks, according to histology and Col2 immunohistochemistry (Hao et al., 2010). Interestingly, the cell density of implanted hydrogels was very high (4 × 10^7^), although it was not discussed as an effective parameter on cartilage formation. In another study, chitosan–glycerol phosphate (GP) was implanted in horse joints to apply high mechanical forces on the hydrogel (Martins et al., 2014). While the hydrogel was biocompatible after 180 days, and cells synthesized Col2 and proteoglycans, Col1 was not assessed.

Several studies used various natural and synthetic materials to fabricate composite constructs to enhance biological properties of the constructs (Filová et al., 2007; Filova et al., 2008). For example, a composite hydrogel of fibrin and HA with autologous chondrocytes was implanted in the knees of miniature pigs, and results were dependent on the initial cell densities, with a lower cell density showing better histological parameters (Rampichová et al., 2010). Immunohistochemistry of Col2 yielded positive staining at the borders of the defects, whereas the center of the defects was characterized by fibrocartilaginous tissue based on the histology scores and low Col2 staining. Injecting a cell-impregnated hydrogel into a porous scaffold has been a useful method for the fabrication of hybrid composites with enhanced mechanical properties (Filová et al., 2007). For example, chondrocytes impregnated into the fibrin hydrogel or an MPEG-PLGA scaffold/chondrocyte/fibrin composite were compared with an untreated defect or an untreated defect with a microfracture intervention (Lind et al., 2008). The composite construct had the best cartilage repair, based on the macroscopic appearance and histology scores only, without any assessments for Col1 and Col2. As reviewed in this section, very few studies investigated Col1 vs. Col2 production within implanted hydrogels, and they mostly focused on just histology and analysis of Col2. Only one study reported hyaline cartilage formation based on Col1 and Col2 staining (Dorotka et al., 2005), whereas two studies showed high levels of Col1 staining within the implanted hydrogel constructs (Schagemann et al., 2009; Marquass et al., 2011).

### Biomedical Imaging as Future Tools for Analyzing Implanted Constructs

Post-surgery assessments for hyaline cartilage regeneration are destructive and invasive and require a high number of animals, which makes *in vivo* operations expensive and complicated (Lind et al., 2008; Heiligenstein et al., 2011; Izadifar et al., 2014; Kundu et al., 2015). Alternative methods have been utilized to visualize implanted constructs in a non-destructive and 3D manner, including confocal microscopy, Raman spectroscopy, optical coherence tomography, positron emission tomography, and single-photon emission computed tomography (Huzaira et al., 2001; Müller and Zumbusch, 2007; Ahearne et al., 2008; Nam et al., 2015). However, these techniques have limitations at increasing tissue depth and volume (Appel et al., 2013; Nam et al., 2015).

Micro-computed tomography (micro-CT) is a helpful tool that can visualize implanted constructs and recognize structural details of the constructs as well as surrounding tissues (Izadifar et al., 2016b; Duan et al., 2021; Ning et al., 2021). However, investigation of hydrogel constructs and soft tissues, such as skin, muscle, and cartilage, is relatively challenging with conventional desktop micro-CT because of its poor contrast with low attenuation coefficients from hydrogels and soft tissues (Muehleman et al., 2002; van Lenthe et al., 2007; Zhu et al., 2011). Further improvements, such as utilizing high-atomic-number element probes or other contrast agents, are required to enhance the quality of the images generated from desktop micro-CT scanning (Mizutani and Suzuki, 2012; Pauwels et al., 2013). For this reason, micro-CT has been mostly used to evaluate implanted hard scaffolds in bone to visualize the formation of new bone in the implants (Hedberg et al., 2005; von Doernberg et al., 2006; Renghini et al., 2009).

Micro-CT can visualize formation of calcified cartilage, which is a mineralized tissue of articular cartilage unlike most cartilage that is unmineralized. In one study, cartilage-derived matrix (CDM) scaffolds alone and as a composite scaffold with a calcium phosphate (CaP) base were implanted into osteochondral defects of horse joints, and micro-CT analysis visualized newly formed calcified cartilage and the CaP component of the composite as well as their integration with surrounding native bone (Bolaños et al., 2017). Formation of mineralized tissue was also distinguished using micro-CT in hybrid 3D-printed PCL/fibrin constructs that were implanted in rabbit joints (Shao et al., 2006). Although the degree of bone regeneration could be quantified from the micro-CT data, visualization of cartilage regeneration was not possible. Magnetic resonance imaging (MRI) has been also utilized to characterize regeneration of cartilage after implantation of tissue-engineered constructs. Ramaswamy et al. used MRI to analyze the implanted photopolymerizable poly [ethylene glycol] diacrylate (PEODA) hydrogel within the rabbit chondral defects (Ramaswamy et al., 2008). MRI neither allowed to visualize the amount of the tissue filling within the defects nor could determine whether the implanted hydrogels were maintained within the defects. Transverse relaxation time (T_2_) was measured in this study, and other similar studies, as a parameter to investigate the regrowth of cartilage (Watrin-Pinzano et al., 2004; Keinan-Adamsky et al., 2006; Ramaswamy et al., 2008). Ramaswamy and coworkers found that there was a negative and linear relationship between the MRI T_2_ parameter and the percentage of tissue filling (Ramaswamy et al., 2008). However, a major drawback with MRI scanning is low resolution of the generated images and distinguishing the implanted hydrogel constructs from the surrounding cartilage might be challenging with MRI.

Synchrotron-radiation (SR)–based imaging technique is a novel tool that offers coherent collimated X-rays comprised a high flux of photons that are originated from a storage ring or other type of specialized particle accelerators. Synchrotron X-ray beams with high-energy photons can diffuse through the dense structures such as bone and not only visualize the bony structure but also whatever is being implanted inside the tissue. Fusion of the micro-CT technique and novel SR-based techniques such as phase-contrast imaging (PCI) and diffraction enhanced imaging (DEI) have enabled the imaging of structures such as hydrogels and cartilage that have low absorption coefficients and attenuation contrasts. Micro-CT–SR-based PCI utilizes refraction effects besides the conventional absorption effects to create a phase shift as the X-ray beam propagates through the materials with different X-ray refractive indexes. The phase shift can be observed at edges of the structures, and as a result, the contrast can be enhanced anywhere that the difference in X-ray absorption is weak (Parsons et al., 2008; Elfarnawany et al., 2017). PC X-ray refraction can be more than one thousand times sensitive than the conventional micro-CT absorption contrast (Zhang and Luo, 2011; Appel et al., 2013). Different imaging techniques including SR-radiography, PCI, and DEI were compared at the same energy for imaging of the scaffolds fabricated from poly (lactide) (PLLA) and chitosan under *in situ* conditions of rat muscle tissues. The scaffolds and tissues were not visible with conventional laboratory–based radiography, and PCI could faintly show them. However, DEI images visualized the tissues very clearly, and the scaffolds were distinguished because X-ray scatter could be rejected in DEI (Zhu et al., 2011). 2D and 3D images of printed PCL scaffolds implanted in pig joints *in situ* were also visualized using the DEI technique. Microstructures of the scaffolds were visible, and size of the strands and pores were measurable (Izadifar et al., 2014). 3D-printed hybrid PCL/alginate constructs were imaged in the subcutaneous region of a murine model, and PCI could visualize regeneration of cartilage ECM as well as hydrogel strands *in vitro*. Moreover, degradation of the biomaterials and integration with the host tissues could be evaluated (Olubamiji et al., 2017). However, hydrogel strands are faintly visualized using these techniques, and thus their 3D volume-rendering is challenging.

SR-based imaging as well as MRI techniques have shown a promising ability to visualize low-density structures, and SR-based imaging has been also utilized for *in vivo* experiments too (Bayat et al., 2005; Wang et al., 2020). However, their resolution still needs to be improved to get images with distinguishable components in a 2D and 3D manner. Also, current SR-imaging tools cannot distinguish between different cartilage tissues such as hyaline and fibrocartilage. This ambitious idea requires developments in future research to upgrade the tools and techniques of SR-imaging that would help to scan the animals *in vivo* and find out the type and amount of newly formed cartilages without need for euthanasia of the animals.

### How Mechanotransduction Pathways Regulate Chondrocytes Biochemical Activities

Mechanical loading is sensed by mechanoreceptors on the surface of chondrocytes and transduced for intracellular responses wherein different signaling pathways are initiated, altering gene expression and synthesis of cartilaginous molecules. The process of converting mechanical cues to biochemical reactions is called mechanotransduction. Understanding the underlying mechanism of mechanotransduction is helpful to avoid activation of pathways that would lead to expression of undesired molecules, importantly Col1 for CTE.

Various studies have reported undesirable Col2/Col1 ratios in the ECM of chondrocytes that were subjected to mechanical loadings. Therefore, it is important to understand signaling mechanisms within chondrocytes that increase the expression of Col1 or decrease Col2 expression. Then, a design could be justified to deactivate pathways favoring Col1 expression or activate pathways that stimulate Col2 expression. In this regard, some CTE studies evaluated the effect of mechanical forces on Col1 and Col2 expression but did not include research into how mechanotransduction signaling pathways worked when the tissue-engineered constructs were loaded (Mauck et al., 2000; Wang et al., 2009). However, osteoarthritis research has identified several pathways which are mechanically stimulated in chondrocytes and affect the composition of cartilage ECM, and they will be discussed in the following paragraphs.

#### TGF-β Signaling

In chondrocytes, transforming growth factor-β (TGF-β) signaling is commonly activated by mechanical stimulation and is highly dependent on the composition of surrounding matrix (Zhao et al., 2020), and this has been shown in mechanically loaded cartilage hydrogels (Allen et al., 2012; Bougault et al., 2012). In addition to TGF-β, the pericellular matrix (PCM) enables participation in mechanotransduction by growth factors such as fibroblast growth factor -2 (FGF-2) and bone morphogenetic protein (BMP). This is accomplished through binding of these growth factors to PCM proteins such as heparan sulfate-perlecan (HS-perlecan) followed by release of these growth factors when the PCM deforms due to mechanical stress (Vincent et al., 2007; Tang et al., 2018; Zhao et al., 2020). Two studies of TGF-β signaling in chondrocytes showed that TGF-β signaling upregulates SOX9, an important activator of Col2 expression, and downregulates RUNX2, an important activator of Col1 expression (Chen et al., 2012; Fang et al., 2016). Neither of these studies used mechanical stress to induce TGF-β activity, but the upregulation of a Col2 activator and downregulation of a Col1 activator provide strong evidence that TGF-β signaling can favor hyaline cartilage formation.

TGF-β uses canonical signaling to activate SMAD that both stimulate SOX9 activity and inhibit RUNX2 activity (Chen et al., 2012; Fang et al., 2016). Accumulation of SMAD proteins in the nucleus can lead to induction of HtrA1, a serine protease that breaks down structural proteins in the PCM (Xu et al., 2014), reducing their ability to impact binding of growth factors with their cell surface receptors. Without the PCM acting as a barrier, the tyrosine kinase receptor DDR2 is then able to bind to Col2 in the ECM, which may oppose hyaline cartilage formation. DDR2 can also increase IL-1β, an inflammatory molecule, which may hinder hyaline cartilage formation. In addition, DDR2 is capable of activating RUNX2, an important inducer of Col1, in differentiating osteoblasts and maturing chondrocytes (Hirose et al., 2020). The stimulation of DDR2 as a result of TGF-β signaling, likely caused by elevated mechanical stress, may increase the levels of MMP-13 and RUNX2, therefore breaking down collagen while increasing the synthesis of Col1 and thus opposing the formation of hyaline cartilage. This would be an opposite response to normal TGF-β signaling, which favors hyaline cartilage formation, warranting further research.

In addition, YAP and TAZ are two mechanosensitive proteins that help SMADs accumulate in the nucleus, potentially contributing to the increase in TGF-β signaling (Varelas et al., 2010; Xu et al., 2014); however, this has yet to be observed in chondrocytes. YAP and TAZ might be more active when high levels of mechanical stress prevent their inhibition by Hippo (Zhao et al., 2020). This would mean that the increase in TGF-β signaling is dependent on the level of mechanical stress the cell is subjected to. Taken together, the effects of TGF-β signaling are much more complex than simply favoring the formation of hyaline cartilage and that overactivity is associated with osteoarthritis and cartilage degradation (Bottini et al., 2019).

#### αV Integrin Signaling

Integrins are a large family of transmembrane receptors and are known for their interactions with extracellular proteins. Numerous integrins transduce mechanical stress and are essential for cartilage health (Yanoshita et al., 2018). Two of these integrins, αVβ3 and αVβ5, are of particular interest due to their effects on inflammatory molecules in response to high levels of mechanical stress. Activation of integrins αVβ3 and αVβ5 upregulated MMP-3 and MMP-13, which break down collagen as well as the inflammatory markers IL-1β and TNF-α (Guan et al., 2015). IL-1β has a role in osteoarthritis progression and has also been shown to cause cartilage degradation via induction of ADAMTS-4 and MMP-13 (Lee H. P et al., 2017). This finding is particularly interesting as the study applied slow-stress relaxation to chondrocytes in a 3D alginate hydrogel, meaning this molecular mechanism has been directly studied in the context of CTE and may be affected by the viscoelastic properties of cartilage tissue (Lee H. P et al., 2017). IL-1β not only has a role in osteoarthritis progression and cartilage degradation but also may contribute to fibrocartilage formation because it can induce Col1 expression through induction of the long noncoding RNA *lncRNA-SAMD14-4* (Du et al., 2020).

#### Calcium Signaling

**FIGURE 2:**
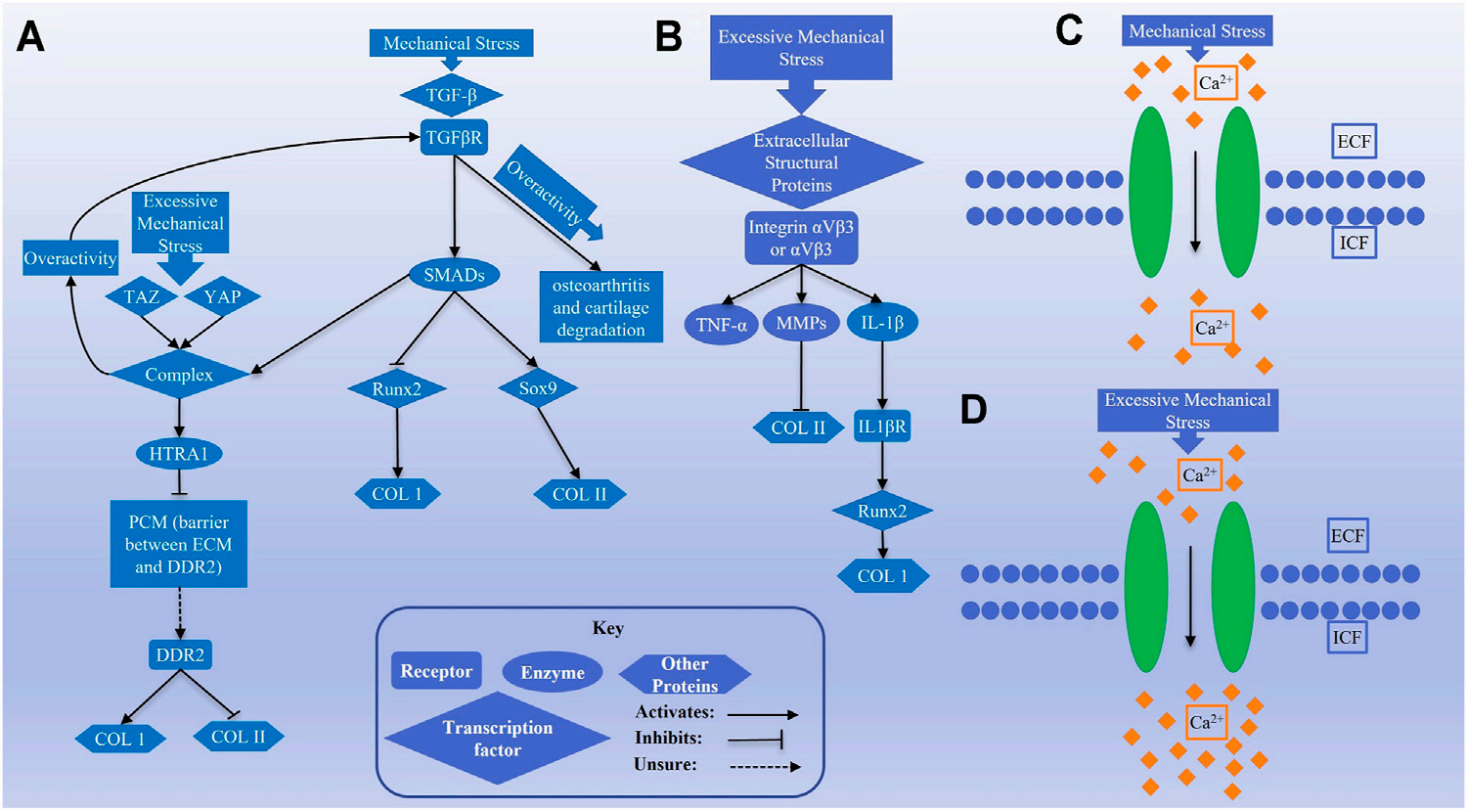
Schematics **(A)** and **(B)** show transforming growth factor-β (TGF-β) and αV integrin signaling pathways, respectively, activated by mechanical stimulation. Schematics **(C)** and **(D)** represent the activity of calcium channels by normal and excessive mechanical stress in chondrocytes.

One of the most essential mechanisms by which cells respond to mechanical stress is through changes in intracellular calcium concentrations, mediated by various mechanically activated channels (Zhao et al., 2020). Transient receptor potential cation channel subfamily V member 4 (TRPV4) is a calcium channel that responds to moderate levels of stress, becoming activated at approximately 3–8% cyclic tensile strain (CTS). Activation of this channel in chondrocytes by mechanical strain caused a moderate level of calcium influx (O’Conor et al., 2014). TRPV4 function can upregulate Col2 and GAG expression, while downregulating the expression of catabolic proteins, such as nitric oxide synthase 2 (NOS2) and a disintegrin and metalloproteinase with thrombospondin motifs 5 (ADAMTS-5), the latter of which functions similarly to matrix metalloproteinases (MMPs) (Wei et al., 2018). This effect on gene expression by TRPV4 favors the formation of hyaline cartilage. The role of TRPV4 in hyaline cartilage maintenance is further supported by the fact that its dysfunction is associated with osteoarthritic characteristics (Agarwal et al., 2021). PIEZO is another calcium channel that responds to mechanical stress but displays properties that differ greatly from those of TRPV4. PIEZO channels respond to excessive mechanical stress, becoming activated at 13% CTS or greater and causing greater calcium influx than TRPV4 channels (O’Conor et al., 2014). Large calcium influx has the potential to become toxic and cause apoptosis and cartilage degeneration (Zhao et al., 2020). This difference between TRPV4 and PIEZO draws parallels to the difference between moderate and overactive TGF-β signaling, where some signaling seems beneficial to the formation of hyaline cartilage but detrimental when it is overactive. A study of TRPV5 in chondrocytes performed by Wei, et al. provided some insight into what mechanisms TRPV5 and PIEZO may exert their effects through (Loeser, 2014). Calcium influx through TRPV5 activates CAMKII, which then subsequently activates AKT, ERK, JNK, and p38 (Loeser, 2014), providing further evidence that calcium signaling through mechanically activated channels such as TRPV4 and PIEZO may affect Col1 and Col2 expression as the TGF-β and αV integrin signaling mechanisms do. Discussed signaling pathways are schematically shown in Figure 2 to illustrate how these pathways are acting in response to mechanical forces.

Although previous osteoarthritis literature provided preliminary evidence of which pathways may be involved in the expression of collagen, future studies of cell signaling in CTE should use 3D culture to determine whether the signaling mechanisms of interest are involved in mechanotransduction and analyze the effects of the pathways on Col1 and Col2 expression. This would provide the field of CTE with potential solutions to the problem of fibrocartilage formation in mechanically stimulated, chondrocyte-impregnated hydrogels. Such solutions could include drugs targeting receptors on the chondrocyte surface, transgenic approaches to silence certain genes, and alternate designs which shield chondrocytes from the applied forces, thereby preventing the overactivity of cellular processes such as calcium, integrin, and TGF-β signaling pathways. Finally, the viscoelastic effects of the stress relaxation rate on cell signaling should be investigated due to the aforementioned ability of the stress relaxation rate to affect IL-1β mechanotransduction. Hydrogel constructs can be designed to have certain viscoelastic properties (Lee et al., 2020), potentially offering another avenue to produce ideal engineered hyaline cartilage.

### 3D-Bioprinted Hybrid Constructs Can Shield Cells From Applied Forces

Hydrogels are soft hydrated networks, and studies have shown that Col1 production and formation of a fibrocartilage-like tissue is the outcome when chondrocyte-impregnated hydrogels are compressed *in vitro* or implanted in a mechanical environment. Thus, mechanical forces are detrimental for chondrocytes in terms of hyaline cartilage formation. Besides, hydrogels generally have low mechanical properties to withstand high compressive forces after implantation in joint defects.

A hybrid construct composed of a cell-impregnated hydrogel loaded within a solid scaffold that is fabricated from a synthetic polymer is an alternative to enhance the mechanical properties of the constructs and reduce the magnitude of applied force on chondrocytes within the hydrogels. Various types of hybrid constructs have been developed and tested *in vitro* for articular cartilage regeneration; however, few of them studied the effects of loading forces on the production of Col1 vs. Col2 (Lee et al., 2005; Grad et al., 2006; Li et al., 2010). Polyurethane scaffolds filled with chondrocyte-impregnated fibrinogen hydrogels were compressed with a joint-kinematic–mimicking regime that was a combination of various compression movements. The gene expression data showed a significant decrease in *Col1a2* expression in the loaded samples compared to unloaded samples. However, Col1 was still present as a thick layer at the upper regions of the loaded samples, whereas it was observed as a thin layer on the upper surface when the constructs did not experience any loading (Wu Y et al., 2017). In another study, an increase in *Col1a2* gene expression happened in loaded fibrin–polyurethane constructs, compared to that in unloaded constructs. Levels of GAGs and *Col2a1* expression in the loaded samples were similar to those in control constructs (Lee et al., 2005). Composite constructs do not prevent production of Col1 from cells when the constructs are subjected to mechanical forces. The reason is that the applied force on a hybrid hydrogel-loaded construct is not shielded from the hydrogel and impregnated chondrocytes, but it is yet shared between the hydrogel and scaffold portions of the construct. Therefore, the cells still sense a high magnitude of the applied forces. Hence, the architecture of a hybrid construct is crucial to provide a load-shielding structure instead of a load-sharing one.

**FIGURE 3:**
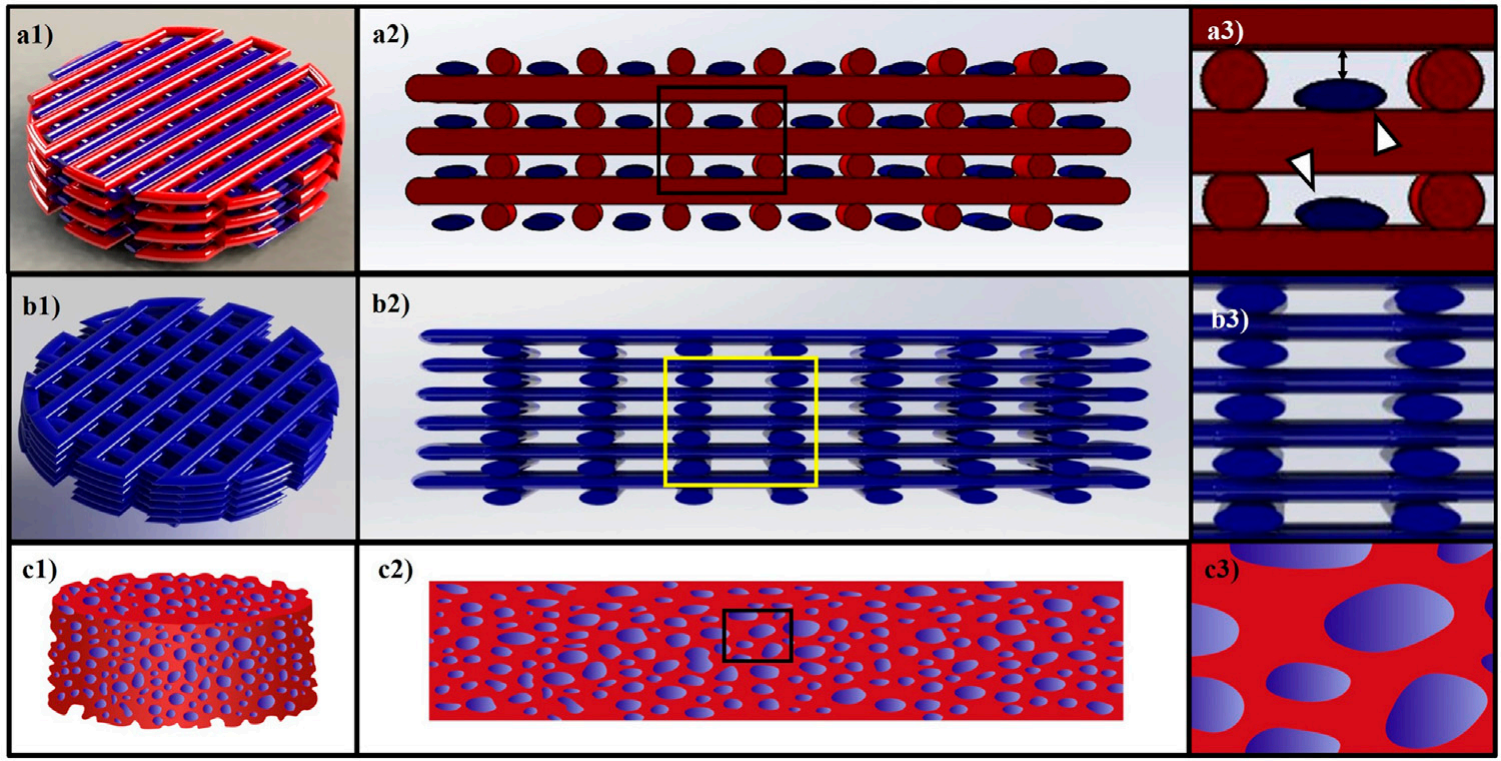
**(A1–A3)** shows a model of a 3D-bioprinted hybrid alginate/PCL construct. Red and blue colors represent synthetic and cell-impregnated hydrogel strands, respectively. White arrows in **(A3)** point to the smaller hydrogel strands that are shielded by the PCL strands when loading is applied. **(B1–B3)** represents a 3D-bioprinted hydrogel construct, and the cells are impregnated within hydrogel strands without any shielding. **(C1–C3)** is a schematic for a hybrid hydrogel-loaded scaffold that cells are within the hydrogel part, and the applied load is shared between the hydrogel and synthetic part of the construct.

Novel methods of scaffold fabrications can help to shield the applied force using new tools, such as 3D-bioprinters, which can fabricate hybrid constructs in a layer-by-layer manner with alternating strands of hydrogel and a synthetic polymer. 3D-bioprinted hybrid alginate–PCL constructs were fabricated with embryonic chondrocytes impregnated within the alginate strands (Izadifar et al., 2016a). These constructs supported cartilage differentiation of the impregnated cells both *in vitro* and *in vivo* (Izadifar et al., 2016a; Olubamiji et al., 2017). In a 3D-bioprinted hybrid construct, the size of the alginate and PCL strands can be adjusted to have smaller alginate strands than PCL and that helps cells in the alginate strands to be shielded from applied forces. The structure of a 3D-bioprinted hybrid construct is depicted in Figure 3 a1-a3, demonstrating how the smaller alginate strands can be protected against the applied forces by the larger PCL strands. Any force-shielding effect only applies to the range of strains when PCL strands do not deform so much that they are the same size as the alginate strands. However, in a 3D-bioprinted hydrogel-only construct, the force is applied on the cell-impregnated hydrogel strands without any shielding effect (Figure 3 b1-b3), thus the cells sensing the total applied force. Figure 3 c1-c3 also represents a hybrid hydrogel-loaded scaffold in which the pores of the scaffold are filled with a cell-impregnated hydrogel. This load-sharing design does not help shield cells because when a force is applied, the force is simultaneously transmitted to the hydrogel and synthetic scaffold because there is not any space separating the hydrogel material from the synthetic scaffold.

## Conclusion and Future Perspectives

Mechanical forces, such as compression loading, can be sensed by chondrocytes within hydrogel constructs, thus affecting the cells’ fates and cartilage regeneration. A major problem in current studies of chondrocyte-impregnated hydrogel constructs that were subjected to compressive force is that the formation of fibrocartilage was not analyzed, but Col1 expression and fibrocartilage-like tissue often can result from such mechanical loading.

Over the past decades, studies employing bioreactors to simulate compression forces in joints illustrated that static compression had limited effects on cartilage regeneration, which has urged researchers to switch to dynamic compression studies. Many *in vitro* dynamic compression experiments did not do specific assessments for Col1 and Col2 together to distinguish the type of newly formed cartilage. However, multiple studies reported upregulation in *Col2a1* gene expression or Col2 deposition in ECM, which would be a good sign for hyaline cartilage formation as long as Col1 expression was not too high also. Loading studies that did assess Col1 production were not in favor of hyaline cartilage regeneration since they showed upregulation of Col1 in response to the loading forces. These results confirmed that high magnitudes of compression forces promoted cells within hydrogels to produce more Col1 thus forming a fibrocartilage-like tissue. *In vivo* implantation of hydrogel constructs also generally reported Col2 production in hydrogel-treated defects of animals. A few of these studies assessed Col1 within the treated defects and reported a high level of Col1 and formation of a fibrocartilage-like tissue.

In conclusion, chondrocyte-impregnated hydrogels subjected to mechanical compression *in vitro* or implanted *in vivo* in joints appear to form a Col1-positive fibrocartilage-like tissue. A possible solution could be to fabricate a chondrocyte-impregnated construct that shields cells from applied forces to avoid Col1 production. 3D-bioprinting and hybrid constructs are appropriate for this purpose, and their application for CTE was demonstrated in recent studies (Izadifar et al., 2016a). *In vitro* compression experiments should investigate the effects of load-shielding in 3D-bioprinted hybrid constructs, looking to exclude Col1 production. Additionally, *in vivo* implantation of hybrid constructs will also help to investigate cartilage formation in response to the various mechanical forces existing in joints.

Cell signaling studies suggest that various signaling pathways may be involved in the expression of collagens within chondrocytes that are activated by normal or excessive mechanical force. More studies of cell signaling pathways in the field of CTE should be performed with fabricated constructs to determine whether the signaling mechanisms of interest are involved in mechanotransduction and to analyze the effects of those pathways on Col1 and Col2 expression. In order to prevent Col1 deposition from mechanically loaded chondrocytes, *in vitro* loading experiments on hydrogels should reveal how to block pathways that direct Col1 expression within the loaded constructs, resulting in more hyaline-like cartilage formation. In *in vivo* studies, SR-based imaging techniques have shown the ability to visualize materials with low attenuation coefficients, such as hydrogels and soft tissues–like cartilage. Also, SR-based imaging tools should be developed not only for enabling quantitative analysis of implanted constructs and newly formed cartilage but also to distinguish between hyaline cartilage and fibrocartilage within a treated defect. Longitudinal SR-based imaging strategies of *in vivo* animal models have begun, so their application to humans is only a matter of time.
